# Immune defects in Alzheimer's disease: new medications development

**DOI:** 10.1186/1471-2202-9-S2-S13

**Published:** 2008-12-03

**Authors:** John R Cashman, Senait Ghirmai, Kenneth J Abel, Milan Fiala

**Affiliations:** 1 Human BioMolecular Research Institute, San Diego, CA 92121, USA; 2 Department of Medicine, Greater Los Angeles Veteran's Affairs Medical Center, Los Angeles, CA 90095, USA

## Abstract

Alzheimer's disease (AD) is a neurodegenerative disease characterized by the accumulation of intracellular and extracellular aggregates. According to the amyloid beta (Aβ) hypothesis, amyloidosis occurring in the brain is a leading cause of neurodegeneration in AD. Defects in the innate immune system may decrease the clearance of Aβ in the brain. Macrophages of most AD patients do not transport Aβ into endosomes and lysosomes, and monocytes from AD patients do not efficiently clear Aβ from AD brain. After stimulation with Aβ, mononuclear cells of normal subjects display up-regulated transcription of *MGAT3*, which encodes β-1,4-mannosyl-glycoprotein 4-β-N-acetylglucosaminyltransferase, and *Toll-like receptor *(*TLR*) genes. Monocytes of AD patients generally down-regulate these genes. A commonly used, naturally occurring material from a spice that enhances certain key functions defective in cells of innate immunity of many AD patients has shown epidemiologic rationale for use in AD treatment. Bisdemethoxycurcumin, a natural curcumin, is a minor constituent of turmeric (curry), and it enhances phagocytosis and clearance of Aβ in cells from most AD patients. We confirmed the effectiveness of a synthetic version of the same compound. In mononuclear cells of most AD patients, bisdemethoxycurcumin enhanced defective phagocytosis of Aβ and increased the transcription of *MGAT3 *and *TLR *genes. The potency of bisdemethoxycurcumin as a highly purified compound in facilitating the clearance of Aβ in mononuclear cells suggests the promise of enhanced effectiveness compared to curcuminoid mixtures. Bisdemethoxycurcumin appears to enhance immune function in mononuclear cells of AD patients and may provide a novel approach to AD immunotherapy.

## Background

Alzheimer's disease (AD) is a major public health problem with a huge associated impact on individuals, families, the healthcare system, and society. It is estimated that as many as five million Americans currently suffer from AD, and 50% of people over the age of 85 may have AD. By the year 2050, the number of affected individuals in the United States is expected to increase to over 13 million [1]. In Europe and other countries, where the number of newborns is decreasing, the number of AD patients is expected to increase dramatically as the population ages [2]. AD is a heavy economic burden on individuals and society, with an estimated annual cost of $100 billion in the US alone. Current therapeutics show only limited effectiveness in ameliorating the symptoms of AD and in improving cognitive ability. Developing an effective therapeutic to combat AD is therefore an immediate and important challenge. Immune-based approaches to treat Alzheimer's disease have shown some promise [3]. However, when applied to humans, immunization with amyloid beta (Aβ) resulted in development of adverse inflammatory responses in a small fraction of the patients tested [4]. Other small molecule immunostimulatory-based strategies may be beneficial. Studies of natural compounds that improve certain defects in innate immune cells of some AD patients suggest a novel and safe therapeutic approach. For example, the natural product mixture curcuminoids selectively enhanced Aβ phagocytosis and gene transcription in blood cells of AD patients [5]. Characterization of the immunostimulatory properties, and the different cellular and gene responses to curcumins, may help to explain observed differences in Aβ phagocytic response between AD and normal individuals, and may eventually lead to diagnostic testing for disease susceptibility or drug response.

## Treatment of Alzheimer's disease

Treatment of AD remains a challenging goal due to our incomplete understanding of its pathogenesis. AD is a multi-component disease, and many biological and physiological steps are involved in the eventual pathological condition. Among other symptoms, the disease is associated with accumulation of neurofibrillary tangles and amyloid plaques in brain tissue of affected individuals. According to the 'Aβ hypothesis', the accumulation of abnormally folded amyloid protein in the brain of AD patients is a leading cause of neurodegeneration [6]. The presence of excess Aβ may be a consequence of two possible pathways: an abnormal and toxic accumulation of Aβ; and a defective detoxification mechanism that would ordinarily clear accumulating Aβ. The mechanisms of neurodegeneration resulting from abnormally folded proteins such as Aβ remain poorly understood. With an increasingly aging population, there exists an urgent need for new and more effective therapeutic approaches [7].

Considerable interest exists in the role that the immune system plays in AD pathology. Macrophages and microglia are the innate immune cells responsible for clearance of pathogens and waste products. It has been shown that peripheral blood mononuclear cells (PBMCs) and macrophages of AD patients cross the blood-brain barrier, but are defective in clearance of Aβ in neuritic plaques, and over-express cyclooxygenase-2 and inducible nitric oxide synthase [8]. Resident microglia in AD brain display markers of phagocytic and inflammatory, but not pro-phagocytic, genes [9]. However, in a transgenic mouse model of AD, most microglia invading Aβ plaques are bone marrow-derived, not resident microglia [10]. Thus, the brains of AD patients and transgenic mice appear to display microglial inflammatory responses, together with defective Aβ clearance by blood-borne macrophages. Defects in the innate immune system of AD patients may therefore play a significant role in brain amyloidosis leading to brain inflammation and neurodegeneration. Enhancement of a patient's innate immunity might represent a novel approach to AD therapy.

In addition to defects in both Aβ phagocytosis and clearance of Aβ in AD brain sections, several molecular markers for monocytes and macrophages have been associated with AD [11]. Unlike PBMCs from AD patients, PBMCs from control subjects were found to up-regulate a number of genes, including, among others, *MGAT3 *(β-1,4-mannosyl-glycoprotein-4-β-N-acetylglucosaminyltransferase), and Toll-like receptor (*TLR*) genes, which are crucial for macrophage function. Evidence for a role of mutated *MGAT3 *in transgenic mouse neuropathology has been published [12], and we have reported suggestive evidence for a correlation between defective *MGAT3 *expression and deficient Aβ phagocytosis [11]. Microarray expression studies also identified several genes significantly up-regulated in AD relative to control PBMCs in response to Aβ exposure [11]. What is not clear from these studies is whether normal expression of these genes is necessary and sufficient for efficient Aβ clearance, or whether other genes and pathways are involved. Also, given previous observations that macrophage Aβ phagocytosis is not defective in all AD patients [5], it is unknown to what extent naturally occurring polymorphisms in these important genes may account for differences in Aβ uptake between individuals. In this context, AD pathogenesis may also involve other polymorphic proteins, including amyloid precursor protein (APP), apolipoprotein E, and other proteins in cellular mechanisms, including APP processing [13], oxidative stress [14,15], and neuronal apoptosis [16-18]. Clearly, a better understanding of the genes responding to Aβ accumulation, and their involvement in Aβ phagocytosis and clearance is needed.

## Curcumins as AD therapeutics

**Figure 1:**
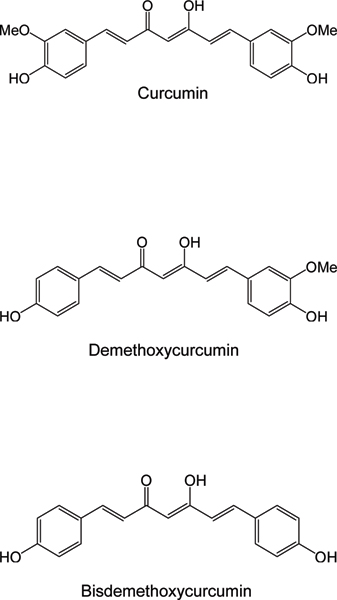
Chemical structures of the principal constituents of curcuminoid. Shown are the three predominant curcuminoids found in natural mixtures. Curcumin is commonly observed to be the major material, whereas demethoxycurcumin and bisdemethoxycurcumin are relatively minor constituents.

Our studies have led us to examine the family of natural products, curcumins, present in significant concentrations in the spices turmeric and red curry, for which there exists epidemiologic and aging-related rationale for use in AD treatment [11]. There is evidence that consumption of these natural compounds in various Asian populations is associated with a protective effect against AD [19]. Also, curcumin has been shown to enhance brain clearance of Aβ in mouse models of AD [20]. However, curcuminoid mixtures are composed of at least three curcumin compounds. The three most prominent members of this class of natural product include curcumin, demethoxycurcumin and bisdemethoxycurcumin (Figure 1). Curcumin is the major material present in curcuminoids, and demethoxycurcumin and bisdemethoxycurcumin are minor constituents. A commonly observed ratio of curcumin:demethoxycurcumin:bisemethoxycurcumin in commercially available curcuminoid mixtures is 66:23:11. As described above, the curcuminoid mixture was shown to possess significant immune-stimulating properties. Based on commonly observed biological activities of natural products, we expected the curcuminoid mixture to contain one compound that was more potent than other materials present. We hypothesized that purification of the most biologically potent material could offer a potential drug discovery path for a new therapeutic approach. We conducted a bio-directed purification of the active component and showed that fractions enriched in bisdemethoxycurcumin could improve the deficits in Aβ uptake and clearance by innate immune cells from AD patients (Figure 1) [11]. Bio-directed purification and isolation of the most potent curcumin involved chemical extraction and chromatographic purification, resulting in increasingly pure compounds until the biological activity was associated with one highly purified fraction. This material was quite pure and liquid chromatography-mass spectrometry showed that it was largely bisdemethoxycurcumin. Bisdemethoxycurcumin was examined for its ability to stimulate phagocytosis of Aβ in cells from control and AD patients. Compared to the curcuminoid mixture, and other curcumin components, bisdemethoxycurcumin was the most potent compound at stimulating phagocytosis of Aβ. The enhanced phagocytosis capabilities in the presence of bisdemethoxycurcumin was also generally associated with increases in expression of *MGAT3 *and *TLR *genes in PBMCs from individuals testing positive for AD. Based on mass spectrometric analysis that identified bisdemethoxycurcumin as the most immuno-stimulatory constituent in curcuminoid fractions, we verified the immuno-enhancing effects of bisdemethoxycurcumin by chemically synthesizing the same material and retesting it alongside other highly purified synthetic and natural curcumins. In all cases examined, bisdemethoxycurcumin showed the most potent immuno-stimulatory activity compared with other synthetic or natural curcumins or demethoxycurcumin. Thus, these studies identified a naturally occurring compound with the potential of at least partially correcting cellular deficits associated with phagocytosis of Aβ of relevance to AD. Because bisdemethoxycurcumin has been used safely (albeit in a mixture of other curcuminoids) for thousands of years, it is possible that it may be directly useful as an agent to treat AD. However, safety studies may be required before the purified compound is administered to humans. Regardless of the advantages and disadvantages of bisdemethoxycurcumin as an anti-AD agent, it can provide a lead molecule for the elaboration of other compounds to be tested as immuno-stimulating agents. The relative ease in synthesizing a variety of derivatives based on the bisdemethoxycurcumin structure allows us to address whether close structural analogs may afford more drug-like compounds and provide enhanced effectiveness and more promising drug candidates.

Extracts containing curcuminoids are currently in clinical use for a number of applications, including treatment of colon and pancreatic cancers, multiple myeloma, AD, and ulcerative colitis [21,22]. Curcuminoid extracts are also available over-the-counter as herbal supplements. Relatively large doses of curcuminoids are being used (that is, 2–8 g/day) because of limited bioavailability. Low bioavailability is likely the result of rapid metabolism and low gut penetration into the bloodstream. For example, curcuminoids compete for conjugation metabolic pathways [23,24], oxidative metabolic pathways [25], and intestinal efflux [26]. To improve clinical utility, and enhance bioavailability, combination formulations involving the use of agents that compete with metabolism and efflux mechanisms likely will be required to increase blood levels of curcuminoids. Regardless, the apparent interaction with oxidative and conjugative metabolic pathways suggests that herb-drug interactions could pose a problem. The approach to identify the most potent constituent in the curcuminoid mixture could circumvent a number of the bioavailability issues if the prominent curcumin components are responsible for drug metabolism shortcomings.

## Defective phagocytosis of Aβ in AD

AD amyloidosis may be related to defective brain clearance of Aβ by monocytes and macrophages and differences in gene transcription [5]. As described before [11], macrophages of most AD patients do not efficiently transport Aβ into endosomes and lysosomes, and AD patient monocytes do not efficiently clear Aβ from the AD brain *in vitro *and *in vivo*, although they retain the ability to phagocytose bacterial pathogens, for example, *Escherichia coli *and *Staphylococcus aureus*. In contrast, macrophages of normal subjects efficiently transport Aβ to endosomes and lysosomes, and control monocytes clear Aβ in AD brain sections. As described more fully in a later section, treating PBMCs from control subjects with Aβ was associated with a relatively elevated expression of the gene *MGAT3*, and genes encoding TLRs. In contrast, and in general, monocytes from AD patients markedly down-regulate these same genes with the same treatment. These levels of expression may correlate with the cells' ability to take up and degrade Aβ, and experimental inhibition of *MGAT3 *expression using small interfering RNA was shown to interfere with Aβ clearance [11].

## Endocytosis and intracellular transport of Aβ in macrophages

Fluorescein isothiocyanate (FITC)- Aβ phagocytosis by macrophages from 42 control subjects, and 73 AD patients, showed that macrophages of control subjects usually (that is, approximately 90%) showed either excellent or extremely efficient phagocytosis of soluble FITC-Aβ within 24 hours. In contrast, a small fraction (15%) of macrophages from AD patients showed extremely efficient phagocytosis and the remainder displayed either minimal surface uptake of FITC-Aβ (60%), or strong surface localization but no intracellular uptake (25%). In macrophages from control subjects, the intracellular transport of Aβ was rapid. After one and two hour post-exposure of macrophages from control subjects, FITC-Aβ co-localized with the early endosomal marker Rab5. In contrast, Rab5 staining and co-localization were minimal in macrophages from AD patients. In macrophages from control subjects, FITC-Aβ co-localized with the lysosomal marker Lysotracker at each time interval (that is, 1, 48, and 72 hours) post-exposure. In contrast, Aβ bound to the cell surface of macrophages from AD patients, but did not progress to lysosomes over a 72 hour period, and the lysosomes were poorly expressed. It appears that the mechanism involving phagocytosis of bacteria is distinct from the phagocytotic mechanism for Aβ.

## Clearance of Aβ in Alzheimer brain sections by control and AD monocytes

As described previously, the phagocytic ability of monocytes to digest Aβ in brain tissue was tested by co-culturing human PBMCs with frozen sections of brain from patients positively identified as having AD [11]. PBMCs from AD patients and controls were co-cultured with frozen serial sections of the frontal lobes of AD patients. The brain section cultures were done under conditions that excluded the role of antibodies in the clearance of brain Aβ. The results were consistent: one-third of the monocytes examined from control subjects were saturated with fluorescently labeled Aβ after 2 days of co-culture, and 100% were saturated after co-culturing for 4 days. In contrast, less than one-quarter of the monocytes examined from AD patients became saturated with Aβ, and the remaining monocytes from AD patients were shrunken and aggregated into clusters with staining that suggested monocyte aggregation and Aβ release. The results suggested that monocytes from control individuals were markedly more effective at clearing Aβ when co-cultured with brain tissue than monocytes from AD patients that were defective in Aβ clearance [11].

## Transcriptional dysregulation in PBMCs of AD patients

Whole-genome microarray analysis of mRNAs was carried out to compare expression patterns in PBMCs of AD patients and controls after overnight treatment with Aβ. Compared to cells from AD patients, control cells up-regulated (>2.2-fold) the transcription of 35 genes and expressed sequence tags (ESTs), including *MGAT3 *(327-fold, *P *< 0.001), and the genes encoding fibronectin (*FN1*; 10.1-fold, *P *< 0.001), cholinergic receptor, muscarinic 4 (9.3-fold, *P *< 0.001), and 2'-5'-oligoadenylate synthetase 3 (*OAS*; 7.8-fold, *P *< 0.001), and down-regulated (>2-fold) the transcription of an additional 35 genes or ESTs. The most prominent transcriptional change in AD macrophages involved down-regulation of *MGAT3 *transcription upon Aβ stimulation. The product of the *MGAT3 *gene is *N*-acetylglucosaminyltransferase III (GlcNAc-TIII), which transfers the bisecting *N*-acetylglucosamine to the core mannose of complex *N*-glycans [27,28]. Another function of GlcNAc-TIII is to stop further processing and elongation of *N*-glycans [29]. GlcNAc-TIII modulates cell interactions, and potentiates α1 integrin-mediated neuritogenesis [30]. Animals with truncated, inactive GlcNAc-TIII show neurological dysfunction [12]. Compared to macrophages cultured from control individuals, macrophages cultured from AD patients are more susceptible to apoptosis by Aβ [15,31]. However, over-expression of GlcNAc-TIII has been shown to protect cells against hydrogen peroxide-induced apoptosis [32], and thus could help stabilize AD macrophages during Aβ phagocytosis. Transcriptional dysregulation of GlcNAc-TIII could also lead to the altered *N*-glycosylation observed in AD [11]. In the AD patients examined, the *MGAT3 *gene defect was strongly associated with AD in the 60–80 years age group, but not in subjects >80 years old. Also, the observed marginal association between decreased *MGAT3 *expression and deficient Aβ phagocytosis (*P *= 0.069) supports the idea that AD subjects >80 years old are distinct in so far as phagocytosis is concerned.

To confirm the suggested transcriptional differences observed between cells from AD patients and control subjects, *MGAT3 *responses to Aβ in PBMCs of 18 AD patients, and 9 control subjects, were investigated using quantitative real-time PCR (qPCR) [11]. Cells from most AD patients (12/18) showed down-regulation of *MGAT3 *(that is, ratio = 0.00001 to 0.99). The data were expressed as a ratio of qPCR signal for the treated versus untreated cells to account for variable baseline values between subjects. The remaining samples from six AD patients (including three AD patients >80 years old) up-regulated *MGAT3*. In contrast, with the exception of two subjects >80 years old, most samples from control individuals up-regulated *MGAT3 *upon Aβ stimulation. Excluding the >80 year-old population samples, in which greater variation was observed, the mean log *MGAT3 *RNA score of AD patients (-1.747) and that of control subjects (+3.77) differed even more significantly (*P *= 0.001). Transcriptional down-regulation in macrophages from AD patients of two other genes noted in the microarray analysis, *FN1 *and *OAS*, was also confirmed by qPCR. Co-treatment with bisdemethoxycurcumin was associated with significant increases in *MGAT3 *gene expression ratios in AD patient cells.

## Transcription of *TLR *genes in mononuclear cells of AD patients and control subjects

TLRs are crucial for macrophage function, and play an important role in the detection of non-self by the innate immune system. Activation of *TLR*s results in many functional outcomes, including enhancement of apoptosis, secretion of inflammatory cytokines, and direct antimicrobial activity [33]. Expression of *TLR *genes in response to Aβ stimulation was examined using PBMCs of AD patients and control subjects. A striking difference was observed in *TLR *mRNA levels between PBMCs from control individuals and AD patients stimulated with Aβ. Whereas control PBMCs exposed to Aβ up-regulated *TLRs*, transcription of *TLR1*,*TLR2*,*TLR3*,*TLR5*,*TLR8*, and *TLR10 *was significantly down-regulated in similarly treated AD cells. It is possible that the lower expression levels of *TLR *genes in AD macrophages may be indicative of more global innate immune defects beyond Aβ phagocytosis.

## Expression profiling

**Figure 2:**
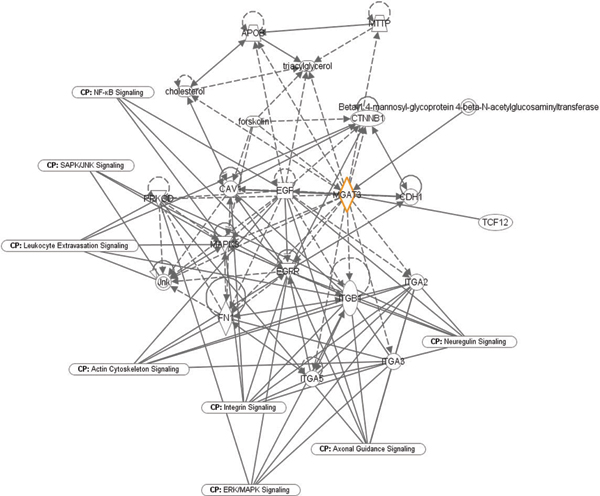
Putative biochemical pathways involving *MGAT3*. Shown are pathways and gene networks with reported interactions involving *MGAT3 *under various conditions. Pathway analysis was performed using IPA tools (Ingenuity). Interactions are shown by lines: lines with arrows represent direct interactions and lines without arrows indicate binding only. Solid lines show reported direct interactions and broken lines show indirect interactions. Functions are indicated by shapes: enzymes (diamonds), cytokines (squares), kinases (triangles), transcription regulators (horizontal ovals), transmembrane receptors (vertical ovals), transporters (trapezoids), complexes (concentric circles), and other (circles).

The microarray and qPCR expression profiling identified certain genes dysregulated in AD relative to control PBMCs in response to Aβ treatment. We sought to identify gene networks and biochemical pathways that may be significantly up- or down-regulated in response to Aβ and curcumins. Such gene pathway analyses may help identify additional targets to understand the complexity of AD as well as to find new potential targets for therapeutic intervention. We used *in silico *tools from Ingenuity  to identify pathways reportedly involving *MGAT3 *that may be associated with the phagocytic and transcriptional responses dysregulated in AD patient cells. Figure 2 depicts other gene products and pathways for which interactions involving *MGAT3 *in various cell types, and under various conditions, have been reported. Based on other proteins in the network, it is interesting to speculate that *MGAT3 *in PBMCs may be part of signaling pathways involving integrins plus Jun- and MAP-kinases, and may contribute to leukocyte extravasation into tissues, among other biological activities. Integrating the pathway information with microarray data from differently treated cells will allow the assembly and quantification of sets of genes that appear to be significantly over-represented among all known genes, and determine if those genes are related biologically. In this way, we can determine which metabolic, biochemical, and transcriptional networks and pathways appear to be responsive to Aβ and curcumin treatments. An important objective of the pathway analyses will be to investigate the roles of these, and their possibly novel activities, in the context of Aβ phagocytosis in PBMCs. These analyses may reveal other responding genes in addition to *MGAT3 *and the *TLR *genes, possibly suggesting other cellular targets that may be amenable to drug intervention.

## Conclusion

Mononuclear cells from AD patients generally display defective phagocytosis and transcriptional down-regulation of certain key genes associated with phagocytosis. Compared with age-matched mononuclear cells from controls, cells from AD patients showed significant defects in innate immunity, including poor clearance of Aβ *in vitro *and marked differences in gene transcription. After Aβ stimulation of mononuclear cells from AD patients, the most prominent transcriptional defect observed involved down-regulation of *MGAT3 *and TLR genes. *MGAT3 *may play a pivotal role in Aβ phagocytosis because silencing of its transcription inhibited Aβ phagocytic function in monocytes from control individuals. *MGAT3 *encodes an enzyme (GlcNAc-TIII) that modulates cell interactions on a number of levels through *N*-glycosylation of key proteins. It is possible that such an over-arching enzymatic activity that is responsible for regulating a whole host of biological processes is a lynchpin in the complex process of phagocytosis. Undoubtedly, an array of coordinated events involving a large number of proteins and receptors (many of which require *N*-glycosylation for their biological activity) is required for Aβ phagocytosis, and *MGAT3 *appears to play a prominent role. Another gene family that is significantly down-regulated in monocytes from AD patients stimulated with Aβ is the *TLR *family of genes, members of which are crucial for macrophage function. TLRs are pattern recognition receptors, found on many cell types, including cells of the innate immune system, that recognize conserved pathogen associated molecular patterns. Activation of TLRs results in many functional outcomes, including enhancement of apoptosis, secretion of inflammatory cytokines and recognition of foreign materials, including bacteria. Thus, TLRs perform a number of global functions for innate cellular immunity. A defective TLR system is anticipated to have far-reaching consequences for abnormal Aβ phagocytosis as well as other functions in innate immunity beyond Aβ phagocytosis.

Our studies led us to examine the natural product curcumins, present in significant concentrations in the spices turmeric and red curry, as there is an epidemiologic and aging-related rationale for their use in AD treatment. There is evidence that consumption of these natural compounds in various Asian populations is associated with a protective effect against AD [19]. Also, curcumin has been shown to enhance brain clearance of Aβ in mouse models of AD [20,34]. Offering the potential for a new therapeutic approach, we identified a certain constituent of this natural compound mixture (that is, bisdemethoxycurcumin) that can improve the defects in Aβ uptake and clearance by the innate immune cells from AD patients [11]. Administration of bisdemethoxycurcumin to mononuclear cells from AD patients was generally associated with enhancement of Aβ phagocytosis and an increase in expression of *MGAT3 *and *TLR *genes in most AD patients. Stimulation of mononuclear cells from control individuals with bisdemethoxycurcumin did not change Aβ phagocytosis nor increase expression of *MGAT3 *or *TLR *genes, suggesting that the curcuminoid bisdemethoxycurcumin was selectively up-regulating key genes in cells from AD patients. We verified the effects of the immuno-enhancing compound (bisdemethoxycurcumin) using a chemically synthesized version. Thus, these studies led to the identification of a natural compound with the potential of correcting cellular defects associated with AD. Synthesis of more drug-like derivatives of bisdemethoxycurcumin may allow us to address whether structural analogs could provide enhanced effectiveness and provide promising drug candidates. A more complete knowledge of the gene expression profiles in innate immune cells in response to biologically active compounds will be invaluable toward understanding the mechanisms of Aβ uptake, and for guiding rational drug development efforts.

## List of abbreviations used

Aβ: amyloid-beta; AD: Alzheimer's disease; APP: amyloid precursor protein; MGAT3: β-1,4-mannosyl-glycoprotein 4-β-N-acetylglucosaminyltransferase; PBMC: peripheral blood mononuclear cell; TLR: Toll-like receptor.

## Competing interests

A provisional US patent has been submitted by JRC and MF involving curcuminoids as therapeutics, and genetic markers associated with Aβ phagocytosis as useful diagnostics.
